# Unraveling the Immunopathogenesis and Genetic Variants in Vasculitis Toward Development of Personalized Medicine

**DOI:** 10.3389/fcvm.2021.732369

**Published:** 2021-09-21

**Authors:** Bryan Ju Min Yap, Ashley Sean Lai-Foenander, Bey Hing Goh, Yong Sze Ong, Acharaporn Duangjai, Surasak Saokaew, Caroline Lin Lin Chua, Pochamana Phisalprapa, Wei Hsum Yap

**Affiliations:** ^1^School of Biosciences, Taylor's University, Subang Jaya, Malaysia; ^2^Biofunctional Molecule Exploratory Research Group (BMEX), School of Pharmacy, Monash University Malaysia, Bandar Sunway, Malaysia; ^3^College of Pharmaceutical Sciences, Zhejiang University, Hangzhou, China; ^4^Unit of Excellence in Research and Product Development of Coffee, Division of Physiology, School of Medical Sciences, University of Phayao, Phayao, Thailand; ^5^Center of Health Outcomes Research and Therapeutic Safety (Cohorts), School of Pharmaceutical Sciences, University of Phayao, Phayao, Thailand; ^6^Unit of Excellence on Clinical Outcomes Research and IntegratioN (UNICORN), School of Pharmaceutical Sciences, University of Phayao, Phayao, Thailand; ^7^Unit of Excellence on Herbal Medicine, School of Pharmaceutical Sciences, University of Phayao, Phayao, Thailand; ^8^Division of Pharmacy Practice, Department of Pharmaceutical Care, School of Pharmaceutical Sciences, University of Phayao, Phayao, Thailand; ^9^Division of Ambulatory Medicine, Department of Medicine, Faculty of Medicine Siriraj Hospital, Mahidol University, Bangkok, Thailand; ^10^Centre for Drug Discovery and Molecular Pharmacology (CDDMP), Faculty of Health and Medical Sciences (FHMS), Taylor's University, Subang Jaya, Malaysia

**Keywords:** vasculitis, autoimmune disorder, immunopathogenesis, susceptibility loci, personalized medicine

## Abstract

Leukocytoclastic vasculitis (LCV) is a systemic autoimmune disease characterized by the inflammation of the vascular endothelium. Cutaneous small vessel vasculitis (CSVV) and anti-neutrophil cytoplasmic antibodies (ANCA)-associated vasculitis (AAV) are two examples of LCV. Advancements in genomic technologies have identified risk haplotypes, genetic variants, susceptibility loci and pathways that are associated with vasculitis immunopathogenesis. The discovery of these genetic factors and their corresponding cellular signaling aberrations have enabled the development and use of novel therapeutic strategies for vasculitis. Personalized medicine aims to provide targeted therapies to individuals who show poor response to conventional interventions. For example, monoclonal antibody therapies have shown remarkable efficacy in achieving disease remission. Here, we discuss pathways involved in disease pathogenesis and the underlying genetic associations in different populations worldwide. Understanding the immunopathogenic pathways in vasculitis and identifying associated genetic variations will facilitate the development of novel and targeted personalized therapies for patients.

## Introduction: Vasculitis Epidemiology and Classification

Vasculitides are a group of multi-system diseases characterized by inflammation of blood vessels, endothelial injury and tissue damage (1). Referring to the Chapel Hill Consensus Conference (CHCC) nomenclature system, vasculitides are classified according to the size of the affected vessels, lesion histopathology and other clinical findings (2). Leukocytoclastic vasculitis (LCV) refers to a type of small vessel vasculitis, where it can be characterized based on several histopathological findings including presence of neutrophil infiltrates, leukocytoclasis (fragmented nuclear debris from neutrophils), fibrinoid necrosis, and damaged vessel walls at the affected vessels (3, 4). In this review, we focus on two forms of LCV, namely the cutaneous small vessel vasculitis (CSVV) which describes small vessel vasculitis that is usually confined to the skin, and anti-neutrophil cytoplasmic antibody (ANCA)-associated vasculitis (AAV), which is usually a severe and systemic condition. Currently, there is a lack of consensus on whether AAV should be classified as a form of CSVV or be treated as a distinct form of vasculitis; in this review, however, we discuss these two forms separately. Both CSVV and AAV are of interest because their incidences have been steadily increasing over the years, most likely due to greater awareness in clinicians and having more definitive diagnostic criteria for each condition. Insights from this review can bridge gaps in knowledge for the development of personalized medicine to treat these two types of vasculitis in patients who do not respond to conventional therapeutic strategies.

CSVV is the most common type of vasculitis in dermatology, mainly affecting the post-capillary venules of the skin (5). The incidence of CSVV ranges between 15 and 38 per million/year, with a prevalence between 2.7 and 29.7 cases per million people (6). In the United States, a population-based study determined an incidence of 4.5 per 100,000 person-years in biopsy-proven cases of LCV (7). The trigger for CSVV may either be idiopathic or due to defined causes such as medications, infections and underlying rheumatologic diseases (8). These vasculitides often involve superficial dermal vessels and manifest as purpuric macules, petechiae or hemorrhagic vesicles and urticarial lesions mainly on the lower legs (5, 8). The cutaneous manifestations are sometimes associated with burning sensations, itchiness or pain (9). However, there is a lack of evidence to say that CSVV impairs the mobility and mobility of affected individuals. CSVV can be diagnosed using skin biopsy, based on the presence of pathological features of LCV when evaluated histologically. However, these features of LCV are found in different subtypes of CSVV such as cryoglobulinemic vasculitis, IgA vasculitis (Henoch-Schonlein purpura, HSP) and hypocomplementemic urticarial vasculitis (anti-C1q vasculitis, HUV), as well as in other forms of vasculitis (6). Hence, apart from using histological findings, specific diagnosis must be accompanied by the evaluation of clinical features by clinicians. While cutaneous signs of CSVV are sometimes accompanied by systemic symptoms such as fever, joint and muscle aches, systemic progression and multi-organ inflammation is not seen and if present, often requiring differential diagnosis for other systemic vasculitides, such as AAV (5).

AAV is characterized by microvascular endothelial inflammation, leading to extravascular inflammation, progressive injury, tissue destruction, fibrosis and loss of function in affected tissue (1). AAV is classified as a rare disease, with an estimated historical prevalence of 48–184 cases per million people (1). However, in the past 30 years, the incidence and prevalence of AAV have increased, with increased peak age of onset and geographical variations in female-to-male incidence ratios (10). More recently, the global prevalence rate has been reported to be 300–421 cases per million persons (1). Increased number of AAV cases may be attributed to factors such as having better classification criteria and definitions, greater awareness amongst clinicians, improved patient survival and prognosis, and wider availability of serological assays for diagnosis (1, 10). AAV is diagnosed by the presence of ANCA targeting perinuclear myeloperoxidase (p-ANCA) and cytoplasmic protease-3 (c-ANCA) (11, 12). Several subtypes of AAV have been identified, including microscopic polyangiitis (MPA), granulomatosis with polyangiitis (GPA, formerly Wegener's granulomatosis), and eosinophilic GPA (EGPA, formerly Churg-Strauss syndrome) (2, 11, 13).

## Vasculitis: Immunopathogenesis and Alterations in Cellular Signaling Pathways

### Cutaneous Small-Vessel Vasculitis (CSVV)

**Figure 1:**
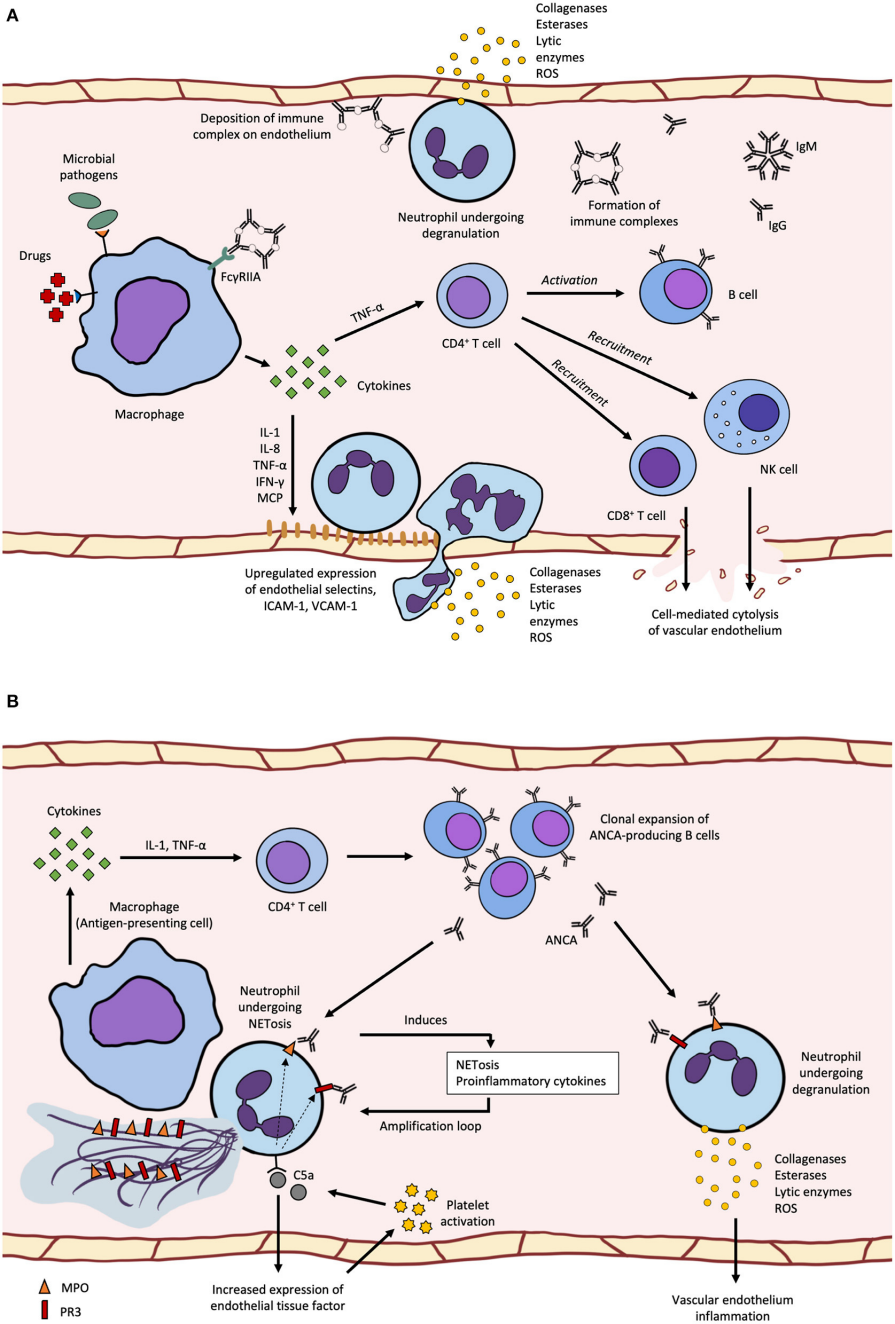
**(A)** cutaneous small-vessel vasculitis (CSVV) begins with pre-exposure to microbial pathogens or certain drugs can induce pro-inflammatory cytokine secretion by macrophages leading to increased endothelial selectin, ICAM-1 and VCAM-1 expression, neutrophil diapedesis and degranulation that damages the vascular wall and surrounding tissues. Activation of the adaptive immune system leads to recruitment of cytotoxic lymphocytes and production of IgG and IgM immune complexes that deposit along the endothelium and induce neutrophil degranulation; **(B)** ANCA-associated vasculitis (AAV) can be caused by APC recognition of surface or secreted neutrophil autoantigens, MPO and PR3, which leads to the production of anti-neutrophil cytoplasmic antigen antiboodies (ANCAs). ANCA can bind to primed neutrophils expression MPO or PR3 on the surface and induce degranulation or NETosis, which forms an amplification loop of antigen release and recognition. Activation of the alternative complement pathway through C5a-mediated upregulation of tissue factor and platelet activation also forms an amplification loop, where C5a binding on neutrophils results in migration of cytoplasmic MPO and PR3 to be displayed on the cell surface.

The major pathogenetic mechanism in CSVV is the Gell and Coombs type III immune complex-mediated reaction (5) (Figure 1A). The latent period between the trigger and manifestation of CSVV can range from seven days to more than 2 weeks, depending on the time required to produce sufficient quantities of antibodies and antigen-antibody complexes upon encountering a stimulus (14). Immune complexes (ICs) circulating in the patients can activate the complement system, generating C3a and C5a anaphylatoxins, which initiate neutrophil chemotaxis and release of vasoactive amines causing endothelial cell retraction (5). Pro-inflammatory cytokines and chemokines, such as IL-1, TNF-α, IFN-γ, IL-8, MCP-1, and RANTES produced by macrophages increase the expression of endothelial selectins, intercellular adhesion molecule-1 (ICAM-1), and vascular cell adhesion molecule-1 (VCAM-1), which promotes neutrophil diapedesis. After the neutrophils exit the blood vessel, neutrophil degranulation releases collagenases and elastases, and generates reactive oxygen species (ROS), resulting in sustained inflammation and fibrinoid necrosis of neighboring vessel walls (5). ICs can also deposit directly on endothelial cells and trigger localized inflammation, such as the deposition of IgA-ICs in glomerular capillaries as seen in cases of Henoch-Schonlein purpura with glomerulonephritis (5). As the disease progresses, lymphocytes gradually become more abundant over time and may also be involved in the pathogenesis and disease progression, eventually becoming the most abundant cell type in lesion histopathology (4). CD4^+^ helper T cells secrete cytokines, notably IL-1, IFN-γ, and TNF-α, and recruit CD8^+^ cytotoxic T cells, B cells and natural killer cells to the affected site, further promoting inflammation (5). Unlike in systemic vasculitides such as GPA, CSVV is not associated with abnormal circulating Treg counts, indicating that CSVV usually does not have systemic involvement (4).

Previous studies have identified various stimuli that can trigger the production of pathogenic antibodies in CSVV patients, which subsequently lead to immunopathology. Hepatitis C virus infection can induce the production of cryoglobulins (immunoglobulins that precipitate at temperature below 37°C), leading to cryoglobulinemic vasculitis where cryoglobulin immune complexes precipitate and deposit on affected small vessels. This subsequently activates the classical complement pathway and causes endothelial damage (15). Besides infection, several drugs have also been known to trigger cutaneous hypersensitivity vasculitis, often presenting as superficial neutrophil or lymphocytic small vessel vasculitis. Drug-induced CSVV accounts for ~20% of all CSVV cases (16). Examples of drugs that may induce CSVV include TNF-α inhibitors and levamisole (17, 18). CSVV may also be triggered directly by endothelial damage due to infections by vasculotropic viruses such as the COVID-19 causative agent, SARS-CoV-2 (14); or indirectly by antibodies generated against exposed autoantigens, such as antiphospholipid antibodies and anti-neutrophil cytoplasm antibodies (ANCA) (5). Although possible, ANCA are rarely found in patients with CSVV, with cases being defined in a distinct subgroup as ANCA-associated vasculitides (AAV).

### ANCA-Associated Vasculitis (AAV)

Although the pathogenesis of AAV is multifaceted and multifactorial, it shares many aspects of that in CSVV, involving the activation of both cytokine-primed neutrophils and alternative complement pathways (11) (Figure 1B). ANCAs are autoantibodies generated by the immune recognition of autoantigens, such as neutrophil myeloperoxidase (MPO), proteinase-3 (PR3) and neutrophil elastase (NE) (19). While most MPO and PR3 are localized in the cytoplasm of unstimulated neutrophils, small amounts of these antigens can be found on the cell surface, even in healthy individuals. However, healthy individuals have only low levels of circulating natural autoantibodies against these auto-antigens, and the epitope specificity of their MPO-ANCA differs from that of pathogenic MPO-ANCA (2). In AAV patients, several mechanisms such as apoptosis and NETosis (release of neutrophil extracellular traps) can further promote the release of cytoplasmic antigens into the extracellular space or increase cell surface expression of cytoplasmic antigens by neutrophils. It was shown *in vivo* that neutrophils from AAV patients with active disease were more likely to undergo apoptosis and their cells had higher expression of surface PR3 and MPO (20). In kidney biopsies from AAV patients, NETs comprising DNA, histones, granule proteins MPO, PR3, LL37, and NE were found in the glomeruli (20). In addition, levels of inflammatory mediators such as tumor necrosis factor-alpha (TNF-α) and C5a were increased in AAV patients and these can also promote the migration of intracellular MPO and PR3 from the cytoplasm to the cell membrane or into the extracellular space (21, 22).

ANCAs play various roles in the pathogenesis of AAV, contributing to disease development and progression. They are able to induce NETosis by binding to FcγRIIA on neutrophils and their ability to induce NETosis correlated with disease activity (20, 23). ANCAs can also bind to cytoplasmic antigens exposed on neutrophil surface leading to respiratory burst, neutrophil degranulation and release of inflammatory mediators such as pro-inflammatory cytokines, ROS and lytic enzymes, which can damage the vascular endothelium (11, 24). ANCA-activated neutrophils can induce injury in nearby microvascular beds, with NETosis leading to the release of neutrophil autoantigens for presentation by antigen-presenting cells. In individuals with active disease, increased numbers of defective Tregs and Bregs may be found (25, 26). Functional Bregs are potent immunosuppressors and inducers of Tregs, where Breg deficiency has been demonstrated in various autoimmune diseases such as SLE and MS, and most likely in AAV (26). The loss of an exon-2-deficient FOXP3 in Tregs due to aberrant splicing results in the loss of downstream protein sequestration involved in immunosuppressive pathways disrupts adaptive immune tolerance, leading to activation of neutrophils and subsequent inflammation of the vessel walls (1, 2, 11, 25, 27). Defective Treg function is thought to be associated with exaggerated neutrophil activity, although the underlying mechanisms are not fully understood (28).

In a mouse model of MPO-ANCA vasculitis, neutrophil depletion prevented disease progression, highlighting the pivotal role of neutrophils in the development of AAV (11). In AAV patients, there is a higher proportion of autoantigen-presenting neutrophils, which can trigger the production of ANCAs through effect T cell recruitment and subsequent B cell activation, resulting in disease (1). Circulating neutrophils isolated from patients with active disease have been shown to generate more basal superoxide, suggesting that they have been primed *in vivo* (24). In addition, surface expression of PR3 on apoptotic cells acts as a signal to initiate efferocytosis by macrophages. PR3-expressing apoptotic neutrophils increase production of pro-inflammatory cytokines, chemokines and nitric oxide (NO) via the IL-1R1/MyD88 signal pathway (11). The internalization of PR3 results in diminished anti-inflammatory macrophage reprogramming, leading to sustained inflammation in a positive feedback loop (29).

Activation of the complement system through the alternative pathway also has a central role in the development of AAV, bridging the inflammatory and coagulation processes found in active disease (30). C5a induces expression of tissue factor (TF) on neutrophils and endothelial cells, triggering the extrinsic coagulation pathway. Increased expression of TF, which may result in hypercoagulability, has been reported in AAV patients with active disease (31). Various components of the coagulation and fibrinolytic cascades can cleave C3 and C5 to generate C3a and C5a, respectively. Activated platelets express receptors for C3a and C5a, while C5b-9 induce the release of alpha-granules and microparticles which further activate the complement system. Platelet counts are often elevated in patients with active AAV and these correspond to disease severity, though the actual crosstalk and interactions are not fully understood (30). In a mouse model of MPO-ANCA vasculitis, C5-deficient mice were completely protected from developing the disease (32), suggesting that C5a can be a potential therapeutic target.

Although less common, eosinophilia has also been identified in cases of AAV, most commonly in EGPA (33). EGPA is characterized by three phases: (1) asthma and allergy symptoms, (2) tissue and blood eosinophilia, and (3) necrotising vasculitis (33). The presence of ANCAs have been reported in 30–40% of EGPA patients (33). While the direct pathogenic mechanisms of eosinophils in EGPA are unclear due to a lack of a suitable animal model, it has been considered to be a T_H_2-mediated disease. CCL17 (a chemokine that recruits T_H_2 cells) and IL-25 (a cytokine that induces and enhances T_H_2 responses) are amongst the mediators that have been implicated in EGPA pathogenesis (33).

## Genetic Variants and Their Association With Immunopathogenesis in Vasculitis

The human leukocyte antigen (HLA) region, also known as the major histocompatibility complex (MHC), is a region within the human genome with the highest density of genes that encode for several important molecules involved in immune responses, and has been frequently associated with autoimmune diseases (34). HLA molecules encoded by this region present autoantigens to T cells, resulting in the development of pro-inflammatory or suppressive T cells depending on how the autoantigens are presented, subsequently leading to either autoimmunity or protection from disease (35). Antigen specificity is determined by specific pockets in the antigen-binding groove of the HLA molecule, and therefore the amino acid sequence of these pockets is crucial to understanding the risk for certain autoimmune diseases (35). While the MHC constitutes the strongest association in vasculitis, there are several loci outside the MHC, including *SERPINA1, PRTN3, SEMA6A, PTPN22, CTLA4, FCGR3B*, and *ARPC1B* that have also been established as genetic risk factors for these diseases, coding for immunological molecules that increase susceptibility to autoimmunity (36) (Table 1).

**Table 1:** Relationship between vasculitis immunological mechanisms and susceptibility loci in different populations.

**Type of vasculitis**	**Immunological target**	**Susceptibility loci**	**Associated population**	**Mechanism**	**References**
ANCA-associated vasculitis	HLA-DP	DPB1^*^04:01, DPB1^*^04:02, DPB1^*^23:01	European	The risk haplotype allele underpins a β chain polymorphism in the antigen-binding pocket of HLA-DP, influencing T cell allorecognition.	(37)
	HLA-DQ	DQA1^*^03:02, DQB1^*^03:03	Chinese	DQA1^*^03:02 codes for Asp160 on the DQ *α* chain. Asp160 forms a salt bridge with His111 on the DQ β chain, stabilizing the HLA homodimer and better priming the CD4+ T cells.	(38, 39)
	HLA-DR	DRB1^*^09:01	Japanese	Binds to MPO and forms an MPO/HLA-DR complex to transport the MPO protein to the cell surface. Exposed MPO associated with HLA-DR on cell surface induces autoantibody production.	(40)
	α1AT	*SERPINA1* (rs28929474/null allele)	European	Inhibits PR3 and thus inhibits inflammatory responses induced by PR3. Under-expression of the gene causes increased levels of circulating PR3, resulting in synthesis of PR3-directed ANCA.	(37, 41–43)
	PR3	*PRTN3* (rs62132293)	European	A proteinase which leads to proteolytic vessel damage. Risk variant causes overexpression of gene by neutrophils.	(37, 41, 43, 44)
	SEMA6A	*SEMA6A* (T allele of rs26595)	European	Involved in the immune response related to autoimmune disorders.	(42, 45, 46)
	Lyp	*PTPN22* (rs2476601)	European	Base substitution at Lyp620W makes Lyp more susceptible to proteolytic degradation, impairing its inhibitory effects on T cell activation and reducing immune tolerance.	(37, 47)
	CTLA-4	*CTLA4* (rs231726, G allele of rs3087243, rs3096851)	European	Individuals with the risk variant (G allele of rs3087243) have T cells with lower activation threshold and thus an increased risk for autoimmune diseases.	(42, 48)
	CD16B	*FCGR3B* (NA1, NA2)	Caucasian	Engaged by anti-PR3 antibodies to activate the ANCA-effector response, causing respiratory burst, phagocytosis, and neutrophil degranulation, thus affecting the severity of disease.	(49)
Cutaneous small vessel leukocytoclastic vasculitis	ARPC1B	*ARPC1B*	Inconclusive	Homozygous complex frameshift mutation causes under-expression of the gene. ARPC1B deficiency in neutrophils and platelets leads to defects in Arp2/3 actin filament branching, resulting in blood and immune-related diseases.	(50–52)

### Genetic Variants Associated With Cutaneous Small-Vessel Vasculitis

Currently, there is not much literature on the genetic factors that contribute to CSVV pathogenesis. However, some have suggested that the gene *ARPC1B* may play a role in predisposition to this disease (36, 37). The *ARPC1B* gene encodes ARPC1B, which is an isoform of ARPC1 (actin-related protein complex-1) and one of the regulatory subunits of the Arp2/3 complex involved in actin polymerization and cellular motility (35). Genetic defects in the proteins regulating cytoskeletal rearrangements often cause syndromes involving the blood and immune systems (50). Arp2/3, in particular, is believed to have a critical role in immune cell synapses formation and T-regulatory cell function (51). A homozygous complex frameshift mutation in *ARPC1B* was identified in a 7-year-old Moroccan boy who presented with a novel combined immunodeficiency involving recurrent infections, mild bleeding tendency, vasculitis (including leukocytoclastic vasculitis), and allergic reactions (52). This mutation had resulted in a complete lack of the ARPC1B protein in the boy's neutrophils. Kahr et al. also described three patients suffering from CSVV who showed homozygous mutations in the *ARPC1B* gene (51). As a result of this mutation, complete loss of or minimal ARPC1B protein in their platelets led to defects in Arp2/3 actin filament branching associated with a range of diseases including inflammatory diseases and cutaneous vasculitis. Thus, the *ARPC1B* gene may be identified as a possible susceptibility locus contributing to CSVV pathogenesis.

### Genetic Variants Associated With ANCA-associated Vasculitis

#### MHC Associations

With regards to the HLA region, the alleles DPA1, DPB1^*^04:01, DPB1^*^04:02, DPB1^*^23:01, DQB1^*^02, and DRB1^*^03 were associated with the development of PR3-ANCAs, whereas DQA1^*^03:02, DQB1^*^03:03, and DRB1^*^09:01 were associated with the MPO-ANCAs (38, 53). These risk factors were identified based on previous association studies (35, 37, 38, 53, 54).

Merkel et al. found a strong association between the *DPB1* risk haplotype allele with AAV in European populations, suggesting that the allele gives rise to a β chain polymorphism in the antigen-binding pocket of the HLA-DP molecule, which may impact T cell allorecognition and thus affect susceptibility to autoimmune disease (37). Furthermore, the association between DQA1^*^03:02 and DQB1^*^03:03 with MPO-ANCA pathogenesis that was found in Chinese subjects may be due to the formation of stable HLA class II *αβ* homodimers (38). These stabilized homodimers facilitate CD4+ T cell priming, whereby CD4+ T cells can only be primed if an immune complex is formed, made up of the stabilized *αβ* homodimer, T cell receptor, and the antigen (39). The *αβ* homodimer is stabilized through the formation of a salt bridge between Asp160 on the DQ *α* chain, encoded by the risk allele DQA1^*^03:02, and His111 on the DQ β chain (38). On the other hand, the role of DRB1^*^09:01 in AAV pathogenesis for the MPO-ANCA subgroup in Japanese populations has been attributed to the association between MPO proteins and HLA-DR molecules encoded by DRB1^*^09:01 (40). A major role of HLA class II molecules is to present antigens to T cells, where HLA-DR, a type of HLA class II molecule, had a high affinity to MPO. HLA-DR binds to intracellular MPO to form an MPO/HLA-DR complex in order to transport the MPO protein to the cell surface, thus initiating the production of autoantibodies against this complex (40). This suggests that MPO associated with HLA-DR are structurally different from native MPO, likely due to cryptic autoantibody epitopes on MPO being exposed by binding with HLA-DR. These structurally different MPO proteins are recognized as “neo-self” antigens by immune cells, therefore inducing autoantibody production (55).

#### Non-MHC Associations

A susceptibility locus associated with AAV targeting PR3, a proteinase causing proteolytic vessel damage, was identified as *SERPINA1* with haplotype rs28929474 in European populations (37, 41). This gene encodes for α1-antitrypsin (α1AT), an inhibitor of PR3 and PR3-induced inflammatory responses (37). As for the risk variant, it causes under-expression of the gene, suggesting that it leads to increased levels of circulating PR3, thus resulting in the synthesis of ANCA directed to PR3 (41). Furthermore, the gene *PRTN3* (haplotype rs62132293) encoding PR3 was identified as a susceptibility locus for AAV in individuals of European descent, with the risk variant causing overexpression of this gene by neutrophils and thus increased expression of PR3 (37, 41).

Another susceptibility locus is *SEMA6A* (rs26595 T risk allele), found to be significantly associated with GPA in a genome-wide association study involving subjects of European descent (42). The functions of semaphorin 6A, encoded by this gene, are not well-characterized, and its role in the risk for GPA remains unclear, but a possible link points to the involvement of semaphorins in the immune response in autoimmune disorders (42, 45, 46).

Meanwhile, the gene *PTPN22* with haplotype rs2476601 has been associated with AAV pathogenesis in European populations due to the link between the risk variant to the aberrant increase in dendritic cell activation and lymphocyte antigen receptor signaling (37). The risk variant encodes Lyp620W which leads to dendritic cell and lymphocyte hyper-responsiveness, increasing the risk for autoimmune diseases (47). Lyp (lymphoid-tyrosine phosphatase) downregulates T cell antigen receptor (TCR) signaling, and risk variants are associated with multiple autoimmune diseases including rheumatoid arthritis (47, 56, 57). Lyp620W has a loss-of-function effect; at the site of the Lyp620W variant, the arginine to tryptophan substitution causes Lyp to become more susceptible to proteolytic degradation, reducing Lyp levels and impairing its inhibitory effects on T cell activation, thus compromising immune tolerance (47). However, the mechanism by which this loss-of-function effect of the Lyp620W variant impacts AAV pathogenesis has yet to be identified.

*CTLA4* was confirmed as a genetic risk factor in CSVV (48). Three haplotypes of this gene—rs231726, the G allele of rs3087243, and rs3096851—were identified in European populations (42, 48). *CTLA4* encodes the protein CTLA-4 (cytotoxic T lymphocyte antigen 4), expressed on activated T cells, which represses T cell activation by associating with CD80 and CD86 on antigen-presenting cells (48, 58, 59). CTLA-4 competes with CD28, a T cell co-stimulant, for CD80 and CD86 binding, and CTLA-4 levels increase when T cells are activated via T cell receptor and CD28 (58). Patients with GPA were found to have increased levels of CTLA-4, a sign of T cell activation (60). Steiner et al. suggested that elevated levels of CTLA-4 are involved in the development of Th1 cells (the primary T cell subpopulation in GPA), due to the role of CTLA-4 in the differentiation of T cells into Th1 cells in TcR transgenic mice. It is also elucidated that individuals carrying the G allele of rs3087243 possess T cells with a lower activation threshold, thus leading to a higher risk for autoimmune diseases (48). The complete role of *CTLA4* in AAV pathogenesis, however, is not fully understood and requires further investigation (48).

The gene *FCGR3B* could also be considered a susceptibility locus for AAV. The role of its genetic variants, namely NA1 and NA2, in AAV pathogenesis can be explained through their involvement in the ANCA-effector response (49). The ANCA-effector response was associated with inflammatory necrosis of small blood vessels (61). ANCA-induced effector mechanisms are triggered when anti-PR3 antibodies bind to granular PR3 presented on activated neutrophils (49). These anti-PR3 antibodies then engage IgG Fc receptors such as FCGR3B (CD16B), encoded by *FCGR3B*, which further activate the ANCA-effector response, leading to respiratory burst, phagocytosis, and neutrophil degranulation, and this affects the strength of ANCA-induced activation of neutrophils and thus the severity of AAV (49).

## Treatment Strategies for Vasculitis

Due to genetic factors playing a major role in vasculitis pathogenesis, targeting associated genes and their corresponding molecular pathways is the suitable approach. Over the years, monoclonal antibodies have gained much attention as a promising therapeutic strategy for the management of vasculitis. Monoclonal antibodies can be a suitable alternative to the first-line drugs based on glucocorticoids (GC), which have limitations such as GC resistance and severe complications (62). Here, we discuss recent findings obtained from studies on potential therapies for both AAV and CSVV.

### Cutaneous Small-Vessel Vasculitis

Treatment of CSVV is clinically driven, perhaps due to the lack of understanding of the genetic background of this disease. Approach to therapy depends on the etiology and severity of the disease (63, 64). If the underlying etiology can be identified, such as due to an infection or a known drug, eliminating the cause would be the best course of action (64). If the CSVV is a result of a systemic vasculitis (e.g., AAV), treatment will be determined by the severity of internal organ involvement, and will usually require a combination of steroids and an immunosuppressive drug, for example rituximab for the treatment of AAV (64, 65). Idiopathic CSVV, on the other hand, has an excellent prognosis, with 90% of cases resolving within weeks to months of onset (66). Therefore, conservative treatment like bed rest, elevation of lower extremities, warming, analgesics, non-steroidal anti-inflammatory drugs (NSAIDs) and antihistamines can be used to alleviate symptoms such as burning or pruritus (63). If the condition extends to an ulcerative, nodular, or vesicobullous form or it becomes recurrent, additional aggressive systemic medications are necessary (63).

Diet, especially those involving a specific food allergen, is commonly cited as an inciting agent of CSVV, and adjusting to bland, low-antigenic diets is usually recommended to prevent recurrences of the condition (63). With regards to medication, the corticosteroid prednisone is the most widely used treatment for idiopathic CSVV (63, 64). The anti-gout agent colchicine (0.6–1.8 mg per day) has been shown to resolve CSVV within 1–2 weeks (63, 67–69). If colchicine proves ineffective, dapsone, an anti-inflammatory and antineutrophilic agent, can be substituted or added (64). If CSVV is persistent, daily azathioprine, a steroid-sparing agent, may be prescribed (63, 70).

In 2017, a case was reported whereby a woman in her 40s suffering from CSVV, with a history of intermittent purpuric lesions for 20 years, was successfully treated with leflunomide (71). Leflunomide is a pyrimidine synthesis inhibitor and is an inexpensive, effective treatment for psoriatic and rheumatoid arthritis and several types of vasculitis. The patient was reported to remain free of new skin lesions and other cutaneous symptoms following 4 months of leflunomide therapy. Further investigation through prospective clinical trials is needed to assess the efficacy of leflunomide for CSVV treatment.

A recent study explored the role of oxidative stress in CSVV pathogenesis, suggesting that damage to blood vessels so often seen in CSVV could be caused by an imbalance in redox homeostasis (72). Recruitment of neutrophils to the affected tissues triggers ROS production, leading to lipid peroxidation in skin tissues and forming acrolein, a type of reactive aldehyde (73). The study showed that the concentration of acrolein-protein adducts in the skin of small-vessel vasculitis patients is proportional to disease severity (72). Therefore, CSVV might be treated through the mitigation of oxidative stress using antioxidative pharmacologic agents, while disease activity could be assessed using immunohistochemical assessments of acrolein content in the patient's skin, thus allowing for more targeted treatments (72). One such promising antioxidative therapeutic agent is peoniflorin, the main component of total glucosides of peony derived from the root of *Paeonia lactiflora* Pall. (74–76). The results from a study revealed that peoniflorin reversed the oxidative damage in human umbilical vein endothelial cells caused by hydrogen peroxide, hence suggesting that peoniflorin may be a candidate therapeutic strategy for oxidative stress-related vascular diseases (76).

### ANCA-Associated Vasculitis

Based on findings derived from genetic studies, it has been elucidated that ANCA-mediated neutrophil activation and B cells are crucial to AAV pathogenesis, and therefore potential treatments can include B cell-depleting drugs (36, 77). In terms of the induction of AAV remission, rituximab represents a very promising contender (65). Rituximab is a murine / human chimeric monoclonal antibody against CD20, a B cell marker, and was licensed in 2011 for remission induction of AAV (65, 77, 78). During the first phase of an international randomized controlled trial known as RITAZAREM, rituximab was shown to be very effective in reinducing remission in relapsed AAV patients when taken in combination with relatively low doses of glucocorticoids (77).

One of the classical features of AAV is represented by granulomatous inflammatory lesions, which are initiated by macrophages and CD4+ T cells (79). T cell activation requires a costimulatory receptor (CD28) and CTLA-4 acts as a negative regulator of CD28, preventing the binding of CD28 with its ligand, thus blocking T cell activation (80). Abatacept, a disease-modifying anti-rheumatic drug (DMARD), is made up of the CTLA-4 ligand-binding domain and a modified Fc domain derived from IgG1, hence can be a possible therapeutic option for AAV treatment (80). An open label clinical trial involving patients with relapsing GPA showed that abatacept resulted in remission in 80% of patients (81). There is also an ongoing phase III clinical trial (NCT02108860) that aims to assess the efficacy of abatacept in achieving sustained remission in patients suffering from a non-severe relapse of GPA.

Despite the promise of these biological agents as therapeutics for AAV, they are still limited by adverse effects such as infections (81, 82). One study found that severe pulmonary infections were the major infectious complication observed in AAV patients treated with a low dose of rituximab (82). They attributed the B cell-depleting role of rituximab as a cause of these infections and identified renal dysfunction and old age as risk factors of such adverse effects. Given that both rituximab and abatacept are immunosuppressants, it is not surprising that patients treated with these drugs are more vulnerable to infections, and therefore this adverse event should be assessed accordingly during follow-up appointments with the clinicians (81, 82).

Plasma exchange, an effective treatment against thrombocytopenic purpura, a disorder which causes low platelet count, is also considered as a course of treatment for AAV patients (83–85). Plasma exchange may benefit patients by removing pathogenic ANCAs in the plasma, as well as clotting factors involved in the coagulation cascade (85, 86). However, the PEXIVAS trial, the largest study of plasma exchange in AAV, did not show that adjunctive plasma exchange benefits patients with severe AAV (87, 88). Nevertheless, it was suggested that patients with both ANCA and anti-glomerular basement membrane (GBM) antibodies should still be treated with adjunctive plasma exchange. Treatments for patients with anti-GBM disease involve immunosuppression and adjunctive plasma exchange (87). Since both patients with single anti-GBM positivity and double positivity for anti-GBM antibodies and ANCA experience aggressive pulmonary and renal disease, plasma exchange seems appropriate for treating double positivity patients (87).

## Developing Personalized Medicines for Vasculitis

Personalized medicine (PM) seeks to deliver targeted therapies to patients who have otherwise been unresponsive to previous treatments, based on a well-informed understanding of the mechanisms of the disease within the individual patient, and is currently the desired treatment of choice for rheumatic diseases (89, 90). As a primary step toward the success of PM, actionable biomarkers are required to assess disease pathophysiology and the molecular pathways involved (90). One technology advance in the field of PM in terms of pathophysiology characterization is Big Data, a multi-dimensional approach that enables the generation of vast amounts data and involves genomics, proteomics, metabolomics, and epigenetics in areas of epidemiology and healthcare delivery (90–95). An example of Big Data technology is single cell RNA-seq (scRNA-seq) which details the transcriptional products of an individual cell, allowing researchers to define diseases functionally instead of clinically based on signs and symptoms (90–94). As Big Data technology has been applied in rheumatic diseases such as rheumatoid arthritis, systemic lupus erythematosus, and systemic sclerosis (90), it is reasonable to think that Big Data can also be applied in studies on vasculitis pathophysiology. Hence, as mentioned throughout this review, genetic studies play a crucial role in identifying these mechanisms as well as in determining the genetic predisposition of each unique patient.

Literature on the development of PM for CSVV is considerably less compared to that for AAV, possibly due to the lack of publications on the genetic factors that may contribute to the disease. Nevertheless, scRNA-seq has been shown to be a promising strategy in identifying CSVV risk factors. Using scRNA-seq, the distribution of signaling molecules as well as HLA-II molecules in vascular endothelial cells across different tissues could be assessed (59). It was found that HLA-II genes exhibited higher expression levels in vascular endothelial cells from the skin than that from the thyroid, trachea, and brain, perhaps leading to the higher rate of dermal vasculitis in CSVV (59, 69). Furthermore, tofacitinib has been proposed as a possible therapy for CSVV patients in a recent case report (96). Tofacitinib is a Janus kinase (JAK) 3/1 inhibitor used in the treatment of rheumatoid arthritis, which suppresses inflammation by interfering with inflammatory cytokine signaling (5, 97). The case report presented a 29-year-old Chinese woman with a 5-year recalcitrant CSVV, who upon treatment with tofacitinib, achieved complete recovery, possibly due to tofacitinib interfering with the signal transduction of proinflammatory cytokines (96). However, more investigation into the efficacy and safety of tofacitinib in CSVV treatment is greatly needed since this is the first case of its kind.

Currently, AAV treatment is based primarily on organ involvement and severity of disease, as seen in the use of rituximab in the induction of remission and plasma exchange in patients with severe or refractory AAV suffering from severe renal involvement (serum creatinine > 500 μmol/L) (65, 98–100). Over the years, PM in AAV has been gaining attention due to the increasing understanding of its immune pathology (65). This understanding has identified distinct AAV categories based on the immunological markers PR3 and MPO (100). Treating AAV patients based on their ANCA type (PR3 or MPO) may be more effective than treating them based on their clinicopathologic disease definitions (EGPA, MPA, or GPA), due to the stronger correlation between the differences in pathogenesis, genetics, and treatment responses with the ANCA type (101, 102). For example, the PR3-ANCA subgroup is associated with the variants of HLA-DP, α1AT, and PR3, while the MPO-ANCA subgroup is associated with the variants of HLA-DQ (43). Conversely, differences in genetic associations were found to be weaker when AAV patients were grouped following the traditional GPA/MPA classification (100). Overall, further understanding of the immunopathology of ANCA type may provide a basis for PM for AAV, addressing issues related to cost and unnecessary drug toxicity (100, 103).

Hence, treatments focusing on immunological targets in PR3-ANCA and MPO-ANCA subgroups are being investigated, such as anti-CD20 therapy (rituximab) which reduces in AAV patients the levels of ANCA, shown to stimulate the release of pro-inflammatory cytokines and ROS from monocytes and neutrophils (77, 100). Furthermore, treatments that target the pro-inflammatory cytokines IL-17, IL-21, and IL-23, namely anti-IL-12/IL-23, anti-IL-17, and anti-IL-17R treatments, could be effective for AAV therapy as AAV patients show elevated levels of these cytokines (100). IL-17, IL-21, and IL-23 are involved in the development of Th17 cells, and IL-17 and IL-21 are said to assist autoreactive B cells in patients suffering from systemic autoimmune diseases (104). The cytokine B cell activating factor (BAFF), which stimulates PR3- and MPO-specific B cell differentiation into antibody-secreting cells, may also be targeted (105). In a multicenter, double-blind, placebo-controlled trial, anti-BAFF therapy (belimumab) administered to AAV patients in remission did not reduce the risk of relapse (106). Belimumab did, however, maintain remission in patients who were treated with rituximab prior to the trial. Thus, belimumab as a potential targeted treatment for AAV requires further investigation.

Personalized medicine for AAV can be taken a step further with autoantigen-specific or ANCA-specific treatment (100). A study reported a promising strategy using cytotoxic T cells conjugated with a chimeric autoantibody receptor that recognizes antibodies against PR3 or MPO on B cells in AAV patients (107). These cytotoxic T cells would kill PR3- or MPO-specific B cells in the AAV patient. In addition, the use of peptibodies or peptides linked to an antibody backbone to target pathogenic ANCA has also been suggested for the mitigation of self-reactive B cells in AAV patients (108). Of course, the safety and efficacy of these treatments as well as their feasibility as AAV targeted therapies need to be further assessed.

## Conclusion

It is clear that a deeper understanding of the immunopathogenesis of vasculitis and its associated genetic risk factors has allowed for recent developments in treatment and promising ideas for personalized medicine. However, the specific genetic variants which predispose individuals to CSVV as well as their pathogenic mechanisms have yet to be fully explored. Therefore, genome-wide association studies (GWAS) and population studies are necessary to identify risk alleles associated with CSVV, while functional analyses are crucial to pinpoint the direct causal effects of the risk alleles to aid in the development of targeted treatments. Further investigations into the immune pathology of ANCA types and into prospective targeted therapies are also recommended for the optimization of personalized medicine for AAV.

## Author Contributions

WY and CC conceptualized the project. BY and AL-F wrote the manuscript. BY designed the figures. AL-F prepared the table. All authors contributed to manuscript revision, read, and approved the submitted version.

## Funding

This work was supported by the Ministry of Higher Education under the Fundamental Research Grant Scheme (FRGS/1/2020/SKK0/TAYLOR/02/1) awarded to CC, the Ministry of Higher Education under the Fundamental Research Grant Scheme (FRGS/1/2019/SKK08/TAYLOR/02/2) awarded to WY, Taylor's University through its TAYLOR'S RESEARCH SCHOLARSHIP Programme awarded to BY, and Unit of Excellence on Clinical Outcomes Research and IntegratioN (UNICORN) [Grant number: FF64-UoE003], University of Phayao awarded to SS.

## Conflict of Interest

The authors declare that the research was conducted in the absence of any commercial or financial relationships that could be construed as a potential conflict of interest.

## Publisher's Note

All claims expressed in this article are solely those of the authors and do not necessarily represent those of their affiliated organizations, or those of the publisher, the editors and the reviewers. Any product that may be evaluated in this article, or claim that may be made by its manufacturer, is not guaranteed or endorsed by the publisher.

## Abbreviations

AAV: ANCA-associated vasculitis
ANCA: anti-neutrophil cytoplasmic antibody
ARPC1: actin-related protein complex-1
BAFF: B cell activating factor
CCL17: CC chemokine ligand 17
CD: cluster of differentiation
COVID-19: coronavirus disease 2019
CSVV: cutaneous small vessel vasculitis
CTLA-4: cytotoxic T lymphocyte antigen 4
DMARD: disease-modifying anti-rheumatic drug
EGPA: eosinophilic granulomatosis with polyangiitis
FcγR: receptor for Fc fragment of IgG
FOXP3: forkhead box P3
GC: glucocorticoid
GPA: granulomatosis with polyangiitis
GWAS: genome-wide association studies
HLA: human leukocyte antigen
HSP: Henoch-Schonlein purpura
GUV: hypocomplementemic urticarial vasculitis
GBM: glomerular basement membrane
IC: immune complex
ICAM-1: intercellular adhesion molecule-1
IFNγ: interferon gamma
Ig: immunoglobulin
IL: interleukin
JAK: janus kinase
LCV: leukocytoclastic vasculitis
Lyp: lymphoid tyrosine phosphatase
MCP: monocyte chemoattractant protein
MHC: major histocompatibility complex
MPA: microscopic polyangiitis
MPO: myeloperoxidase
MS: multiple sclerosis
MyD88: myeloid differentiation primary response 88
NE: neutrophil elastase
NET: neutrophil extracellular trap
NO: nitric oxide
NSAID: non-steroidal anti-inflammatory drug
PEXIVAS: plasma exchange and glucocorticoids for treatment of ANCA-associated vasculitis
PM: personalized medicine
PR3: proteinase-3
RANTES, regulated on activation: normal T cell expressed and secreted
ROS: reactive oxygen species
scRNA-seq: single cell RNA sequencing
SLE: systemic lupus erythematosus
TCR: T cell receptor
TF: tissue factor
Th: T helper cell
TNFα: tumor necrosis factor alpha
VCAM-1: vascular cell adhesion molecule-1
α1AT: α1-antitrypsin.
